# Imputation of Below Detection Limit Missing Data in Chemical Mixture Analysis with Bayesian Group Index Regression

**DOI:** 10.3390/ijerph19031369

**Published:** 2022-01-26

**Authors:** Matthew Carli, Mary H. Ward, Catherine Metayer, David C. Wheeler

**Affiliations:** 1 Department of Biostatistics, School of Medicine, Virginia Commonwealth University, One Capitol Square, 830 East Main Street, Richmond, VA 23298, USA; carlimm@vcu.edu; 2 Occupational and Environmental Epidemiology Branch, Division of Cancer Epidemiology and Genetics, National Cancer Institute, Rockville, MD 20850, USA; wardm@exchange.nih.gov; 3 School of Public Health, University of California Berkeley, Berkeley, CA 94704, USA; cmetayer@berkeley.edu

**Keywords:** mixture analysis, environment, below detection limit, Bayesian

## Abstract

There is growing scientific interest in identifying the multitude of chemical exposures related to human diseases through mixture analysis. In this paper, we address the issue of below detection limit (BDL) missing data in mixture analysis using Bayesian group index regression by treating both regression effects and missing BDL observations as parameters in a model estimated through a Markov chain Monte Carlo algorithm that we refer to as pseudo-Gibbs imputation. We compare this with other Bayesian imputation methods found in the literature (Multiple Imputation by Chained Equations and Sequential Full Bayes imputation) as well as with a non-Bayesian single-imputation method. To evaluate our proposed method, we conduct simulation studies with varying percentages of BDL missingness and strengths of association. We apply our method to the California Childhood Leukemia Study (CCLS) to estimate concentrations of chemicals in house dust in a mixture analysis of potential environmental risk factors for childhood leukemia. Our results indicate that pseudo-Gibbs imputation has superior power for exposure effects and sensitivity for identifying individual chemicals at high percentages of BDL missing data. In the CCLS, we found a significant positive association between concentrations of polycyclic aromatic hydrocarbons (PAHs) in homes and childhood leukemia as well as significant positive associations for polychlorinated biphenyls (PCBs) and herbicides among children from the highest quartile of household income. In conclusion, pseudo-Gibbs imputation addresses a commonly encountered problem in environmental epidemiology, providing practitioners the ability to jointly estimate the effects of multiple chemical exposures with high levels of BDL missingness.

## 1. Introduction

There are more than 350,000 chemicals and chemical mixtures registered for production and use globally [1]. Chemicals used for commercial purposes have been found in human tissues and in household air and dust samples in varying concentrations [2,3,4], motivating questions as to their impact on human health. Epidemiologic studies have identified environmental chemical exposure as a risk factor in a number of human diseases, including cancer, type 2 diabetes, cardiovascular disease, thyroid disease, and developmental disorders [5,6,7,8,9,10]. Increasingly, investigations into the health impact of chemical exposures highlight the fact that they exist as mixtures of many simultaneous exposures [11,12]. Therefore, epidemiologists have sought to assess the joint impact of chemical mixtures on health outcomes as opposed to estimating chemicals as independent risk factors [13,14,15].

Several statistical methods have been developed for analyzing chemical mixtures that handle the highly correlated data commonly found in chemical mixtures [16], including weighted quantile sum (WQS) regression [17], quantile g-computation [18], and Bayesian kernel machine regression (BKMR) [19]. WQS regression is a two-step process that estimates a single exposure index from part of the data and then estimates the health effect for the exposure index from the remainder of the data. More recently, group index models were developed to allow for multiple chemical groups, where each of the groups can have different magnitudes and direction of association with the outcome [20,21]. There are both frequentist and Bayesian versions of group index models, with Bayesian models being able to estimate all model parameters simultaneously in one step [21,22,23,24].

One of the challenges of mixture analysis not fully accounted for in these methods is the commonly encountered problem of below detection limit (BDL) missing observations. A detection limit (DL) is defined as the lowest chemical concentration that can be distinguished from a concentration of zero with reasonable confidence [25]. These detection limits can vary between chemicals, assay methods, different laboratories, and with laboratory time [26,27]. Concentrations below this limit are not reported, leading to interval-censored distributions. Traditionally, analysts presented with this missing data problem have resorted to ad-hoc substitution methods for imputation, where the BDL is replaced by 0, the DL, or some function of the DL (DL/2 being a common example). Such simple substitution has subsequently been criticized for leading to biased parameter estimates and variances [28,29,30] and for introducing artificial patterns into the original data [31] and therefore is not recommended practice. Various alternative imputation methods that have been developed, such as maximum likelihood estimate (MLE), restricted MLE [32,33], reverse Kaplan–Meier [34], and empirical “robust fill-in” methods [28]. A criticism of these “fill-in” or single-imputation (SI) methods is that imputations are treated as truly observed data without accounting for their variance; however, there is also some evidence that suggests such methods are suitable at lower percentages of BDL missingness [29]. To address this criticism, multiple-imputation (MI) methods, which account for the variance of imputations, have also been developed [29].

Moving to the Bayesian framework, the most straightforward method of imputing missing covariate data is by drawing imputations jointly from a multivariate distribution [35], often a multivariate normal or *t* distribution. A joint distribution can be hard to define, however, when covariates containing missing data are diverse (a combination of continuous and binary variables, for example) or when non-normal models are required. The imputation of BDLs is an instance of the latter, as these bounded variables are best modelled by truncated distributions. One method developed to deal with these difficult covariate groupings is Fully Conditional Specification (FCS), which imputes missing observations one covariate at a time by a univariate conditional distribution conditioned on all other variables in the model. Each variable in the model is cycled through in this fashion until convergence to an assumed but unspecified joint posterior distribution is reached [36].

A common criticism of FCS is the potential for the various univariate conditional distributions to be incompatible, that is, to fail to converge to any joint distribution [37,38]. Incompatibility can result in unsound imputations and biased estimates [39]. Despite these theoretical concerns, FCS has performed well in simulations and has shown to be robust to incompatibility in some scenarios [40]. An alternative imputation method that addresses the issue of potential incompatibility is what we will refer to as Sequential Full Bayes (SFB) imputation [41]. Similar to FCS, univariate conditional distributions for each covariate containing missing observations are used but, in this instance, in order to factorize the joint distribution as a product of all the conditional distributions [42]. In this manner, the joint distribution of the imputation model is specified, avoiding any issues of incompatibility.

The non-Bayesian imputation methods described above have all been applied in the context of mixture analysis. While not recommended, naïve substitutions are still performed [43], likely due to the convenience of these methods. SI methods, which are more theoretically justified than substitution methods but are also relatively easy to implement, are also commonly employed [44,45]. MI procedures are increasingly used in chemical mixture analysis. Single imputation was performed for 10 datasets in a study of non-Hodgkin lymphoma that utilized WQS regression; however, the resulting estimates were not pooled [46]. A Bayesian MI method was later developed specifically for the imputation of BDLs encountered when performing WQS regression [47]. MI procedures have also been developed for BKMR [48] and quantile g-computation [49]. Bayesian imputation methods, by contrast, are not as commonly employed in mixture analysis. One example is found in a 2010 paper by Herring, where BDLs were imputed by a joint distribution specified as a product of marginal and conditional truncated normal distributions in the larger context of regression analyses of chemical mixtures using a nonparametric Bayesian shrinkage prior [50]. Such simultaneous estimation of missing BDL observations along with the main parameters of interest (index effects and their component weights in the case of Bayesian group index regression) is an attractive solution to the BDL problem.

In this paper, the aim was to extend Bayesian group index regression to handle BDL missing data. To accomplish this aim, we implemented four imputation methods in combination with the Bayesian group index model. The first two are statistical methods that utilize FCS: the well-known Multiple Imputation by Chained Equations (MICE) [51], and what we will refer to as pseudo-Gibbs imputation. As its name implies, MICE involves multiple imputation, where many completely observed datasets are generated by FCS, estimates are calculated for each, and they are then finally pooled into a final result. Pseudo-Gibbs imputation, on the other hand, combines the imputation model (FCS) with the health effects model (Bayesian group index regression) in one Gibbs sampler algorithm from which parameter estimates of interest are derived. A third method utilizes SFB imputation. As with pseudo-Gibbs imputation, this imputation model is combined with the Bayesian group index health effects model in the same Gibbs sampler. Finally, in addition to these Bayesian methods, we consider a type of “fill-in” method where missing BDL observations are singly imputed from a truncated log-normal distribution, which we refer to as Prior imputation.

To evaluate the four imputation techniques mentioned above (MICE, pseudo-Gibbs, Prior, and SFB) in combination with Bayesian group index regression, we conducted a simulation study with varying percentages of BDL observations and compared the model performance. We then applied the best performing method to an investigation of the link between the household exposures and childhood leukemia in the California Childhood Leukemia Study (CCLS). The CCLS data are well-suited for such an analysis, as some of the chemical concentrations gathered in this study exhibit high degrees of BDL missingness. The results from this paper will provide practitioners with a method of analysis that can simultaneously impute BDL observations in a reasonable fashion while estimating the association of chemical mixtures to health outcomes.

## 2. Materials & Methods

### 2.1. Bayesian Grouped Index Regression

The Bayesian grouped index model in general form for a binary health outcome $y_{i}\sim Bernoulli\left(p_{i}\right)$ is specified through the log-odds of disease of the *i*th subject as

(1) $$logit\left(p_{i}\right)=\beta_{0}+\overset{K}{\underset{k=1}{\sum }}\beta_{k}\left(\overset{C_{k}}{\underset{j=1}{\sum }}w_{jk}q_{ijk}\right)+z_{i}^{T}\varphi .\;$$

On the left of the equation is the logit of the disease probability $p_{i},$ and on the right are the effects for the intercept $\beta_{0}$; chemical indices $\beta_{k}$, which estimate the health effects for exposure to the *k*th group of exposures; and a vector of covariates $z_{i}^{T}$ with corresponding effects in vector φ. The number of exposures in each of the *K* indices can vary and is denoted by $C_{k}$. For each index, $w_{jk}$ is the weight for the *j*th exposure in the *k*th index and denotes the relative importance of that exposure within the index. The value of each $w_{jk}$ is constrained to be between 0 and 1, and when summed across an individual index must equal 1. For each index, $q_{ijk}$ is the quantile score for the *j*th exposure in the *k*th index for the *i*th subject. Quantiles are used instead of raw chemical concentration data in order to limit the influence of outliers and to standardize the varying concentration scaling of different exposures. The definition of quantiles adopted (e.g., quartiles, deciles) is at the discretion of the user.

Finally, the model is completely specified by the assignment of prior distributions to the model parameters. For any given index, the weights $w_{1k},\ldots ,w_{C_{k}k}$ are assigned a Dirichlet prior with parameters $\alpha_{jk}=\left(\alpha_{1k},\ldots ,\alpha_{C_{k}k}\right)$. This choice of prior ensures that the weights $w_{jk}\in \left(0,1\right)$ and $\sum_{j=1}^{C_{k}}w_{jk}=1$. Each index effect is given a vague normal prior $\beta_{k}\sim Normal\left(0,\tau_{k}\right)$ with precision $\tau_{k}=1/\sigma_{k}^{2}$ and $\sigma_{k}\sim Uniform\left(0,100\right)$. Any covariate effects also receive vague normal priors.

Inference on health effects and relative importance of chemical exposures is done through the joint posterior distribution. Markov chain Monte Carlo (MCMC) is used for model parameter estimation and convergence to the posterior is established using the Gelman–Rubin diagnostic statistic using two chains. Researchers who wish to use the Bayesian grouped index regression model as detailed in this paper may do so using the R package BayesGWQS [22], which implements Bayesian grouped index models using Just Another Gibbs Sampler (JAGS) [52].

### 2.2. Imputation Methods

As discussed above, missing data imputation is any method by which incomplete data are made complete by substitution with artificial or imputed data. The Bayesian methods implemented were chosen because they each take into account the additional variability of imputed observations. MICE does this through pooling multiple imputations, while SFB and pseudo-Gibbs imputation do so by drawing estimates from converged posterior distributions. The final imputation method, Prior imputation, is a single imputation method that was chosen to highlight circumstances where simpler imputation methods perform just as well as more complex ones and circumstances where they are contraindicated.

#### 2.2.1. Multiple Imputation by Chained Equations (MICE)

MICE imputes missing data through a series of what are referred to as “chained equations”. Given a partially observed dataset, it is assumed the outcome and predictors have a multivariate distribution that is completely specified by some unknown vector of parameters. MICE seeks to obtain a posterior distribution for these unknown parameters without explicitly defining the joint distribution of the data. Imputation models are specified in a univariate fashion for each variable in the dataset, where missing values in any given variable are imputed by a conditional distribution conditioned upon all other variables. These are then linked by means of a Gibbs sampler, which iterates through imputations variable by variable until convergence is attained.

In our application to BDL imputation, our data are composed of a binary outcome *y* and all chemical exposures of interest $x_{j}$, where j=1, … , C. We assume a multivariate distribution of these variables is completely specified by θ, a p=C+1 length vector of unknown parameters. We obtain the posterior distribution of θ by iteratively sampling from the following conditional distributions:

(2) $$P\left(y|x_{1},\ldots ,x_{C},\theta_{1}\right)\;P\left(x_{1}|y,x_{2},\ldots ,x_{C},\theta_{2}\right)\;\vdots \;P\left(x_{C}|y,x_{1},\ldots ,x_{C-1},\theta_{p}\right).$$

The chained equations compose the following Gibbs sampler to impute BDLs, which at the *t*th iteration draws

(3) $$\theta_{1}{}^{*\left(t\right)}\sim \;P(\theta_{1}|y^{obs},x_{1}^{\left(t-1\right)},\ldots ,x_{C}^{\left(t-1\right)})\;\;y^{*\left(t\right)}\;\sim \;P\left(y|y^{obs},x_{1}^{\left(t-1\right)},\ldots ,x_{C}^{\left(t-1\right)},\theta_{1}{}^{*\left(t\right)}\right)\;\vdots \;{\;\theta }_{p}^{*\left(t\right)}\;\sim \;P\left(\theta_{p}|x_{C}^{obs},y^{\left(t\right)},x_{1}^{\left(t\right)},\ldots ,x_{C-1}^{\left(t\right)}\right)\;x_{C}^{*\left(t\right)}\;\sim \;P\left(x_{C}|x_{C}^{obs},y^{\left(t\right)},x_{1}^{\left(t\right)},\ldots ,x_{C-1}^{\left(t\right)},\theta_{p}^{*\left(t\right)}\right)$$

where $x_{j}^{\left(t\right)}=\left(x_{j}^{obs},x_{j}^{*\left(t\right)}\right)$ [51]. One challenge specific to applying this method to the imputation of BDLs is that imputations from these conditional distributions could result in imputed values above the LOD of any particular chemical, contradicting knowledge we already have about that particular observation’s value. For these cases, erroneous imputations are “post-processed,” taking imputations above the LOD and re-imputing them by drawing from a uniform distribution $x_{j}^{*}\sim Uniform\left(0,LOD_{j}\right)$.

#### 2.2.2. Prior Imputation

The Prior imputation method utilizes the so called “data block” in JAGS, where variables can be assigned distributions from which single imputations are drawn. These imputed values are subsequently treated as observed data in the MCMC estimation. This is a type of single imputation or “fill-in” method, which avoids the negative characteristics of ad-hoc imputation methods but, because imputation happens only once, does not reflect the variability in the imputation process. There is some evidence, however, that this underestimation of variance is not reflected in parameter estimates when BDL percentage is below 30% [29]. Specific to our application of this method, BDLs were imputed to follow a truncated log-normal prior $BDL_{ij}\sim Lognorm\left(\mu_{j},\tau_{j}\right)$ restricted to values within the range of (0, $LOD_{j}$). Uniform and gamma distributions were assigned for the mean and precision hyperpriors, with mean $\mu_{j}\sim Uniform\left(0,LOD_{j}\right)$ and precision $\tau_{j}\sim Gamma\left(0.01,0.01\right)$.

#### 2.2.3. Pseudo-Gibbs Imputation

The pseudo-Gibbs method imputes missing BDL observations by including them as model parameters in the MCMC along with the health-effects model parameters. This pseudo-Gibbs sampling process is similar to that of MICE, where variables are imputed one at a time, and the variable being imputed at a particular moment is conditioned on all other variables in the model, current to their most recently updated value. However, the pseudo-Gibbs method is a combination of imputation and health effects models, and therefore, the estimated parameters of the health effects model inform the missing data imputations and vice versa. While each BDL observation is estimated as an individual parameter, BDLs from the same chemical share the same chemical-specific prior and hyperprior distributions. These distributions are the same as those detailed for the Prior imputation method; however, the values drawn from them are not single imputations but estimations sampled repeatedly though MCMC. A distribution is estimated, giving full posterior inference. Convergence of the MCMC algorithm is evaluated using the Gelman–Rubin diagnostic statistic.

#### 2.2.4. Sequential Full Bayes Imputation (SFB)

Similar to the FCS imputation model used in MICE and pseudo-Gibbs imputation, the SFB imputation method relies on a sequence of multiplied univariate conditional distributions to express a joint distribution. Again, we take chemical exposures of interest $x_{j}$, where j=1, … , C. Their joint distribution can be written as follows:

(4) $$P\left(x_{1},\ldots ,x_{C}|\theta \right)=P\left(x_{C}|x_{1},\ldots ,x_{C-1},\theta_{C}\right)\;\times P\left(x_{C-1}|x_{1},\ldots ,x_{C-2},\theta_{C-1}\right)\times \ldots \times P\left(x_{2}|x_{1},\theta_{2}\right)\times P\left(x_{1}{|\theta }_{1}\right)$$

where $\theta_{j}\;$ is a distinct vector of parameters indexing the jth conditional distribution, with the set of $\theta_{1},\ldots ,\theta_{C}$ vectors parameterizing the joint distribution [42]. In our application to BDL imputation, these conditional distributions follow a truncated log-normal prior restricted to values within the range of 0 and that chemical’s LOD. Like the pseudo-Gibbs method, the above imputation model is combined with the Bayesian group index regression model to give full posterior inference on all model parameters, including the index effects and weights.

### 2.3. Simulation Study Design

To evaluate the performance of the four imputation methods, we generated chemical concentration data consisting of three groups (with five chemicals in the first group, four in the second, and five in the third) with a binary outcome. Each group contained a single important chemical, which was set by assigning a true chemical weight of 1 to the important chemicals and 0 to nonimportant chemicals, thereby making the total weight for each group sum to 1. The chemical concentrations were given an across group correlation of 0.3 and a within group correlation of 0.7. The correlation structure was specified through a matrix and then converted into a covariance matrix. A mean vector and standard deviation vector were selected to generate the covariance matrix and hence allow construction of the data that were distributed as multivariate normal.

These predictor groupings and outcome were then used in two different signal-strength scenarios. These scenarios differed in the magnitude their index associations, measured in odds ratios (OR). In Scenario 1, the first group had no association with the outcome (OR = 1.0), while the second and third were associated with OR = 0.80 and OR = 1.25, respectively. Scenario 2 was generated in a similar fashion, except the second and third groups were associated with the outcome with OR = 0.67 and OR = 1.50, respectively. The sample size generated for both Scenarios 1 and 2 was 500 observations. BDLs were introduced to the data by eliminating the lowest observation values up to a certain DL, depending on the percentage of BDLs desired. For each scenario, BDLs were introduced at the 10, 30, 50, and 70 percentage levels.

After defining the true exposure effects, we created binary outcomes for case or control status to replicate a case-control study by having a relatively balanced number of cases and controls (50% ± 10% cases) in each iteration of data generation. The binary outcome y was distributed as y~Binomial(n,p) where $p=\frac{1}{1+e^{\eta }}$ and $\eta =\beta_{0}^{*}+\overset{3}{\underset{k=1}{\sum }}\beta_{k}^{*}[\sum_{j=1}^{C_{k}}w_{jk}^{*}q_{ijk}]$, and the star notation indicates true parameter values. As no covariates were used in generation of the data, the term $z^{T}\phi =0$. The number of quantiles used in all simulations was set at four when computing the weighted index for each group (i.e., $q_{ij}=0,1,2,3$). Each simulation was done with 100 data sets.

To assess the relative performance of the three imputation methods, we calculated the mean squared error (MSE), bias, and power on each of the group exposure effects as well as the sensitivity and specificity of identifying chemicals as important or not. We assessed model fit by comparing the deviance information criterion (DIC) of each method and also compared the computation times. When calculating power, we examined the proportion of 95% credible intervals (CIs) of the odds ratios of chemical group associations that did not contain 1.00. We measured sensitivity by determining the proportion of important chemicals that were identified by the models as being important. This was done by determining if the estimated weight of the important chemicals produced by the models was greater than or equal to the threshold $\frac{1}{C_{k}}$. Likewise, we defined specificity as the proportion of the unimportant chemicals that were correctly deemed unimportant by the models. This was determined by checking if the estimated weights of the unimportant chemicals were less than the same threshold of $\frac{1}{C_{k}}$. DIC was defined as $\;DIC=\bar{D}+p_{D}$, where $\bar{D}$ is the posterior mean deviance [53], and $p_{D}$ is the effective number of parameters [54], a measure of model complexity.

### 2.4. Data Analysis

We next applied our chosen imputation method along with Bayesian grouped index regression to an investigation of childhood leukemia in the California Childhood Leukemia Study (CCLS). The CCLS is a population-based case-control study carried out in 35 counties in California, 17 counties in the San Francisco Bay area, and 18 in the Central Valley [55,56]. Between 1995 and 2012, cases ≤14 years old were ascertained within 72 h of diagnosis from nine major pediatric clinical centers in the study area. Using California birth certificate information, controls were matched to cases on the basis of date of birth, sex, Hispanic ethnicity, and maternal race.

The parents of both case and control participants were initially interviewed to gather information about their child’s exposure to suspected leukemia risk factors. Families who had not moved since the child’s diagnosis date (reference date for controls) were interviewed a second time (Tier 2), during which carpet dust samples were collected. The second interview and dust sampling were limited to cases and controls <8 years old at diagnosis to ensure the samples reflected early-life chemical exposure of the child. Case-control matching was not maintained due to residential eligibility criteria and voluntary participation. There were 731 eligible participants (324 cases and 407 controls). Of these, 296 cases (91%) and 333 controls (82%) agreed to participate. Due to insufficient dust or interferences in the chemical analyses, some chemical concentrations were lost, leading to a final 277 cases and 306 controls (*n* = 583) [57].

Dust samples were collected using either a high-volume small surface sampler (HVS3) or a household vacuum cleaner. As previously described in Colt et al. (2008), concentrations of 64 organic chemicals (ng/g dust) were measured using gas chromatography/mass spectrometry (GC/MS) in multiple ion monitoring mode after extraction with three different extraction methods. Nine metals were measured using microwave-assisted acid digestion combined with inductively coupled plasma/mass spectrometry (ICP/MS).

As discussed in Wheeler et al. (2021b), strong correlations (r > 0.6) between many chemicals in the CCLS data do not allow for the use of traditional regression methods. Bayesian group index regression, on the other hand, is well-suited for mixture analyses of such data. Our analysis investigated the association of 67 chemicals (Table S1) with risk of childhood leukemia. Out of the entire CCLS dataset, only chemical exposure variables with at least 20% non-missing observations were included, as past experience has shown that higher levels of missingness contribute negligible information on potential relations with an outcome.

We organized exposures into seven chemical class indices: polychlorinated biphenyls (PCBs), polycyclic aromatic hydrocarbons (PAHs), insecticides, herbicides, metals, the tobacco exposure markers of nicotine and cotinine, and polybrominated diphenyl ethers (PBDEs). The logic of these groupings was that the chemicals share a structural similarity (e.g., PCBs, PAHs, metals) or usage (e.g., herbicides, insecticides). In addition to these chemical exposure indices, we included child’s age, sex, and ethnicity; annual household income; mother’s education level; mother’s age at birth of child; and whether the child lived at the sampling residence since birth as controlling covariates in the model.

We first fit the 7-group exposure model and then evaluated high family income as a potential effect modifier because it was a consistently significant covariate in previous analyses [23,24].To investigate potential effect moderation, we extended the 7-group model to include seven interaction terms between each index and the highest income level. We then conducted a stratified analysis and dichotomized into the highest income bracket ($75,000+) as one level and the lower five brackets ($0–$74,999) as the second.

We chose the method of BDL imputation suggested by the results of the simulation study described above. There were additional, non-BDL missing data in the PBDE chemicals, as they were measured a few years later than other chemicals on a subset of cases (*n* = 181) and controls (*n* = 214) due to insufficient amounts of dust; in total, PBDEs were not measured on 32.2% of Tier 2 participants [58]. These missing observations were imputed in a similar fashion as BDLs, but their log-normal distributions are not truncated. Continuous chemical concentrations (ng/g) were categorized into quartiles for regression. Convergence of all parameters of interest in models were checked via a Gelman–Rubin diagnostic statistic upper CI less than 1.10. We summarized the results using ORs for each chemical index along with 95% credible intervals and forest plots. Within each index significantly associated with the outcome, we assessed the important chemical exposures using the estimated weights.

## 3. Results

### 3.1. Simulation Study

The estimated odds ratios and power for the Prior imputation, SFB, pseudo-Gibbs, and MICE imputation methods for all scenarios are in Table 1. All imputation methods in each BDL scenario performed similarly for null effect parameters, with the exception of SFB and MICE imputation at 70% BDL, where Type I error rates were noticeably lower. For Scenario 1 (lower signal scenario), power was similar for all imputation methods, with pseudo-Gibbs imputation resulting in slightly higher power in the 70% BDL case. This pattern was repeated in Scenario 2 (higher signal scenario), where the difference in power at 70% BDL in favor of the pseudo-Gibbs method was much more apparent. Power was predictably higher in the more strongly associated Scenario 2, with values more than doubling for all imputation methods. In both scenarios, power tended to decrease as BDL percentage increased, with the drop in power most apparent after the 30% BDL case. While the pseudo-Gibbs method was best able to preserve power from decreasing as BDL percentage increased, absolute power in Scenario 1 at 70% BDL reached extremely low levels for all imputation methods.

MSE and bias of the four imputation methods are compared in Table 2. Both MSE and bias remained relatively consistent as the percentage of BDLs grew. Other than a few exceptional instances, the MICE imputation method estimations had the lowest MSE. The differences in MSE were minimal for the 10% BDL case and was one of the instances where another method (pseudo-Gibbs) outperformed MICE. While differences in MSE were never extreme, they tended to be larger at higher levels of missingness. The Prior imputation method often had the next best MSE after MICE. The results for bias were less consistent. In Scenario 1, pseudo-Gibbs imputation tended to have the lowest bias and if not, was a close second. In Scenario 2, however, pseudo-Gibbs imputation was only the least biased for 10% BDL and was at times the most biased imputation method. MICE and Prior imputation were least biased for 30% and 50% BDL but had the highest bias of all simulations done at 70% BDL. SFB and pseudo-Gibbs had the lowest and second-lowest bias for 70% BDL, respectively.

The sensitivity and specificity of important chemical identification calculated for the four imputation methods is presented in Table 3. Sensitivity for both signal strength scenarios was very similar for all imputation methods until the 70% BDL case, where pseudo-Gibbs imputation had consistently larger sensitivity values. Specificity values were very similar across all imputation methods for each combination of signal strength and level of missingness. SFB and pseudo-Gibbs generally performed best by this statistic. Differences in specificity values increased as the percentage of BDLs increased, most notably in Scenario 2. The odds ratios further from OR = 1.00 predictably resulted in higher values for both sensitivity and specificity. In both scenarios, sensitivity and specificity tended to decrease as BDL percentage rose, with the largest decreases occurring between 50% and 70% BDL.

The model fit tended to decrease (lower DIC is better) for all imputation methods as the percentage of BDLs rose (Table 4). There were very slight differences in DIC at low levels of missingness. For Scenario 1, Prior imputation resulted in the best fit, whereas for Scenario 2, pseudo-Gibbs and SFB performed best. For both signal levels, SFB and pseudo-Gibbs had the lowest DIC as BDL percentage increased, and of the two, SFB was slightly better in Scenario 1, while pseudo-Gibbs was better in Scenario 2. These two methods also saw increases in pD as BDL percentages rose, indicating greater model complexity. Of the four methods, MICE saw the largest increase in DIC as BDL percentage rose. Considering runtime, the Prior imputation method was always the fastest running analysis at around 7 min (Table 4). MICE was the next best, with similar but slightly slower runtime (accomplished with parallel computing). The pseudo-Gibbs and SFB methods were the slowest by far, taking nearly nine hours or more to complete at 10% BDL and nearly two days or more at 70% BDL, averaged over 100 datasets.

### 3.2. Application of Pseudo-Gibbs imputation to house dust chemicals in the CCLS

The results of our simulation study indicate that for data with relatively high percentages of BDL observations, the most suitable imputation method is pseudo-Gibbs imputation. As the CCLS data have 23.9% of 67 chemical exposure variables with greater than 50% BDLs (*n* = 16) and 10.4% with 70% or more BDLs (*n* = 7), we applied this method of imputation when performing the following analysis. We first considered the non-stratified analysis. The odds ratios estimated for index effects and covariates are in Table 5. PAHs were the only index found to have a significant association with childhood leukemia (OR = 1.27, 95% CI: 1.01, 1.60). The PCB index was also positively associated with the outcome although this effect was marginally significant (OR = 1.19, 95% CI: 0.96, 1.51). The two most heavily weighted chemicals in the PAHs index were benzo(k)fluoranthene and indeno(1,2,3 -c,d)pyrene, with posterior mean weights of 0.164 and 0.149, respectively. Looking at the forest plot of estimated index means and 95% CIs (Figure S1), we can see PBDEs was the most variable index estimate. Among the controlling covariates, the highest income category and residence since birth were significant and protective.

Our Bayesian group index regression of interaction effects between the chemical indices and the highest income bracket ($75,000 or more) resulted in a significant interaction between income and the metals index (OR = 0.45, 95% CI: 0.24, 0.82). In the subsequent analysis stratified on household income, three chemical indices were found to have significant associations with childhood leukemia risk in the highest income strata (≥$75,000, 107 cases, 159 controls) (Table 6). PCBs (OR = 1.55, 95% CI: 1.04, 2.36) and herbicides (OR = 2.02, 95% CI: 1.005, 3.99) had significant positive associations with childhood leukemia. The herbicide index had the strongest association but was the most variable. The metals index (OR = 0.42, 95% CI: 0.25, 0.69) was inversely associated with childhood leukemia. Of the covariates, residence since birth was significantly inversely associated with risk. The forest plot of the index association estimates and their 95% CIs are presented in Figure S2. Of the four PCB chemicals, PCB 138 had the highest mean posterior weight of 0.31, followed by PCB 180 with a weight of 0.28. Among the herbicides, dacthal had the largest weight (0.51). In the metals index, arsenic was the most highly weighted chemical (inverse association), with a mean posterior weight of 0.37. The specific estimates for the lower income stratum and its forest plot are presented in Table S2 and Figure S3. There were no significant findings in the lower income stratum (<$75,000).

## 4. Discussion and Conclusions

In this paper, we implemented four methods for the imputation of BDL missing data in the context of Bayesian group index regression and conducted a simulation study to evaluate the performance of these methods at two different association strengths (OR = 1.25 and 1.50) as well as at four different levels of BDL missingness (10%, 30%, 50%, and 70%). We found that the relative performance of the methods was similar across the two association strengths and across the 10–50% BDL levels, with some methods slightly outperforming others in certain scenarios judged by some metrics. Notably, the Prior imputation method performed consistently well across metrics in this BDL range. It was at times the best performing method, was rarely the worst, and when not the best performer, was usually competitive.

Clear differences in performance were seen, however, in the 70% BDL range. At such high levels of missingness, pseudo-Gibbs imputation was found to be the preferred method of imputation. A clear advantage of pseudo-Gibbs imputation was that it consistently had more power to detect significant associations than other methods (with power differences of 10% or more in many instances). This superior performance was also apparent in sensitivity. Results were not so clear for specificity, bias, and DIC, where SFB imputation performed slightly better in some instances. While all imputation methods had approximately the same performance as judged by MSE, pseudo-Gibbs imputation was often the weakest method by a slight margin. The greatest weakness of the pseudo-Gibbs method is its runtime. While faster than SFB imputation, it proved to be much slower than either MICE or Prior imputation. Additionally, while pseudo-Gibbs imputation had the highest power in Scenario 1 at 70% BDL, in absolute terms, power was quite low. Detecting lower signal differences at such high levels of BDL missingness would likely require an increase in sample size even when using the pseudo-Gibbs method.

Based on the findings described above, we recommend pseudo-Gibbs imputation for data where the percentage of BDLs approaches 70% and the Prior imputation method for lower percentages. While 70% BDL missing data is an extreme level of missingness to simulate, such percentages are at times encountered in chemical exposure investigations (CCLS being an example), and previous statistical research has been done for BDL missingness at such levels [29,59]. It should be noted that while our simulated datasets had uniform levels of missingness across all chemical exposure variables, this would be highly unlikely to occur in actual practice. While this represents a simplification from real conditions, we believe our results nonetheless offer useful guidelines for determining the most suitable method of BDL imputation. A further limitation of our results is that they are restricted to the particular scenarios simulated. At higher BDL levels, the slow runtime of the pseudo-Gibbs imputation can be justified most clearly by its improved performance in power and in sensitivity. While second to SFB in some metrics, the difference in their performance was negligible. Importantly, although pseudo-Gibbs was relatively slow, the slowest method was SFB, an increase in runtime which is hard to justify by its performance. At lower percentages, Prior imputation offers a computationally efficient and convenient method that produces estimates competitive with the other methods presented.

Our decision to apply pseudo-Gibbs imputation in our analysis of the CCLS data reflects the above observations. While BDL missingness is not uniform across all chemical predictors in the CCLS observational data, many exhibit BDL levels of 50% or more, with some of these extending to 70% or more (chemicals with 80% or more were excluded). In our application of pseudo-Gibbs imputation to the CCLS observational data, we fit a seven-index model and found a positive and significant association between PAHs (OR = 1.27) and leukemia, with benzo(k)fluoranthene (weight = 0.164) and indeno(1,2,3 -c,d)pyrene (weight = 0.149) having the highest mean posterior weights. Previous research of this study population employing single-chemical models have found either significant or borderline significant associations between these two PAHs and childhood leukemia [60]. In stratified analysis of the highest income category and all others, the chemical indices estimated for the high-income strata tended to be larger and have lower variance. Among children from high-income households, PCBs (OR = 1.55) and herbicides (OR = 2.02) were significantly and positively associated with childhood leukemia, while the metals index (OR = 0.42) was significantly inversely associated with risk.

The association of PCBs with leukemia reflects the findings of earlier work. In a previous study of the CCLS cohort, group index regression methods found a marginally significant association between PCBs and childhood leukemia, with PCB 138 contributing the most to the index effect [24]. Single-chemical logistic regression analyses have also found significant positive associations between leukemia and PCB138 as well as between leukemia and summed total PCB concentrations [56]. Similarly, the significant positive association found for herbicides (and the dominance of dacthal within the index) closely mirrors prior analyses of these data done using Bayesian group index regression analysis with a different imputation approach [23] and GWQS regression [24]. Besides these mixture analyses, univariable logistic regression analyses have found similar associations between dacthal and childhood acute lymphocytic leukemia (ALL) risk [57]. The significant negative association observed for the metals index, and for arsenic in particular, have less support from previous research. While arsenic is a well-known risk factor in adult bladder cancer [61], there is little to no evidence of any link between arsenic and childhood cancer, including childhood leukemia [62]. While selection bias cannot be ruled out to explain the negative association in the current paper, further investigation is necessary to understand this association.

In summary, through our comparison of BDL imputation methods in the context of Bayesian group index regression, the pseudo-Gibbs method of imputation performed best under conditions of high BDL missingness, whereas Prior imputation offers a suitable method of imputation at relatively low levels of BDL missingness. These methods and the guidance for their appropriate use allows researchers assessing environmental exposures to more rigorously handle the common problem of BDL missing data. While our application was to chemical exposure missing data, other fields (such as genomics) that frequently encounter such missing observations could also benefit from these methods.

## Figures and Tables

**Table 1:** Estimated odds ratio (OR) and power values for Bayesian group index regression using four different imputation methods.

Parameter	Prior Imputation		Sequential Full Bayes		Pseudo-Gibbs		MICE	
10% BDL	Estimated OR	Power	Estimated OR	Power	Estimated OR	Power	Estimated OR	Power
exp(β_1_) = 1.00	1	0.07	0.999	0.06	0.999	0.05	1	0.06
exp(β_2_) = 0.80	0.818	0.43	0.818	0.43	0.818	0.43	0.818	0.43
exp(β_3_) = 1.25	1.251	0.43	1.251	0.42	1.251	0.44	1.251	0.43
exp(β_1_) = 1.00	0.994	0.05	0.9934	0.04	0.993	0.04	0.994	0.05
exp(β_2_) = 0.67	0.658	0.9	0.658	0.9	0.658	0.9	0.658	0.9
exp(β_3_) = 1.50	1.553	0.91	1.553	0.92	1.553	0.92	1.554	0.92
30% BDL	Estimated OR	Power	Estimated OR	Power	Estimated OR	Power	Estimated OR	Power
exp(β_1_) = 1.00	1.004	0.08	1.001	0.08	1.001	0.08	1	0.06
exp(β_2_) = 0.80	0.816	0.43	0.814	0.43	0.814	0.43	0.819	0.41
exp(β_3_) = 1.25	1.246	0.4	1.254	0.43	1.253	0.43	1.247	0.42
exp(β_1_) = 1.00	0.996	0.05	0.999	0.07	0.996	0.05	0.994	0.05
exp(β_2_) = 0.67	0.662	0.92	0.655	0.92	0.655	0.93	0.664	0.93
exp(β_3_) = 1.50	1.539	0.9	1.552	0.89	1.556	0.9	1.535	0.89
50% BDL	Estimated OR	Power	Estimated OR	Power	Estimated OR	Power	Estimated OR	Power
exp(β_1_) = 1.00	1.002	0.05	1.004	0.07	1.003	0.07	1.002	0.07
exp(β_2_) = 0.80	0.824	0.37	0.828	0.34	0.812	0.4	0.823	0.38
exp(β_3_) = 1.25	1.241	0.39	1.236	0.35	1.253	0.37	1.234	0.34
exp(β_1_) = 1.00	0.995	0.04	0.995	0.03	0.994	0.05	0.991	0.06
exp(β_2_) = 0.67	0.667	0.88	0.664	0.88	0.651	0.89	0.681	0.88
exp(β_3_) = 1.50	1.521	0.87	1.551	0.88	1.557	0.87	1.498	0.86
70% BDL	Estimated OR	Power	Estimated OR	Power	Estimated OR	Power	Estimated OR	Power
exp(β_1_) = 1.00	0.997	0.06	0.992	0.01	0.997	0.06	0.994	0.03
exp(β_2_) = 0.80	0.857	0.2	0.843	0.2	0.81	0.29	0.857	0.18
exp(β_3_) = 1.25	1.209	0.26	1.25	0.28	1.256	0.26	1.184	0.22
exp(β_1_) = 1.00	0.993	0.02	0.979	0.04	0.987	0.05	0.984	0.01
exp(β_2_) = 0.67	0.724	0.68	0.693	0.66	0.655	0.81	0.753	0.6
exp(β_3_) = 1.50	1.425	0.69	1.53	0.74	1.542	0.75	1.356	0.59

**Table 2:** MSE and bias of index effects from Bayesian group index regression using different imputation methods.

Parameter	Prior Imputation		Sequential Full Bayes		Pseudo-Gibbs		MICE	
10% BDL	MSE	Bias	MSE	Bias	MSE	Bias	MSE	Bias
exp(β_1_) = 1.00	0.012	−0.006	0.012	−0.007	0.011	−0.007	0.012	−0.006
exp(β_2_) = 0.80	0.017	0.014	0.017	0.014	0.017	0.014	0.017	0.014
exp(β_3_) = 1.25	0.014	−0.007	0.014	−0.007	0.014	−0.006	0.014	−0.006
exp(β_1_) = 1.00	0.012	−0.012	0.012	−0.012	0.012	−0.013	0.012	−0.012
exp(β_2_) = 0.67	0.015	−0.026	0.015	−0.025	0.015	−0.025	0.015	−0.026
exp(β_3_) = 1.50	0.017	0.027	0.017	0.027	0.016	0.027	0.017	0.028
30% BDL	MSE	Bias	MSE	Bias	MSE	Bias	MSE	Bias
exp(β_1_) = 1.00	0.012	−0.002	0.013	−0.005	0.013	−0.005	0.012	−0.006
exp(β_2_) = 0.80	0.017	0.012	0.018	0.009	0.017	0.008	0.016	0.015
exp(β_3_) = 1.25	0.014	−0.010	0.015	−0.004	0.014	−0.005	0.014	−0.009
exp(β_1_) = 1.00	0.012	−0.010	0.013	−0.008	0.012	−0.010	0.012	−0.012
exp(β_2_) = 0.67	0.014	−0.019	0.015	-0.03	0.015	−0.031	0.013	−0.015
exp(β_3_) = 1.50	0.017	0.018	0.018	0.025	0.018	0.028	0.016	0.015
50% BDL	MSE	Bias	MSE	Bias	MSE	Bias	MSE	Bias
exp(β_1_) = 1.00	0.014	−0.005	0.015	−0.003	0.015	−0.004	0.013	−0.004
exp(β_2_) = 0.80	0.018	0.021	0.021	0.024	0.02	0.006	0.017	0.021
exp(β_3_) = 1.25	0.014	−0.014	0.015	−0.019	0.015	−0.005	0.013	−0.020
exp(β_1_) = 1.00	0.013	−0.012	0.013	−0.012	0.014	−0.013	0.012	−0.015
exp(β_2_) = 0.67	0.015	−0.011	0.015	−0.017	0.017	−0.036	0.013	0.009
exp(β_3_) = 1.50	0.018	0.005	0.021	0.024	0.02	0.028	0.017	−0.010
70% BDL	MSE	Bias	MSE	Bias	MSE	Bias	MSE	Bias
exp(β_1_) = 1.00	0.02	−0.013	0.019	−0.018	0.022	−0.014	0.012	−0.012
exp(β_2_) = 0.80	0.024	0.058	0.024	0.041	0.026	0	0.017	0.062
exp(β_3_) = 1.25	0.018	−0.042	0.021	−0.011	0.019	−0.005	0.016	−0.060
exp(β_1_) = 1.00	0.016	−0.015	0.025	−0.032	0.022	−0.024	0.014	−0.023
exp(β_2_) = 0.67	0.024	0.069	0.023	0.023	0.024	−0.034	0.023	0.112
exp(β_3_) = 1.50	0.025	−0.062	0.031	0.005	0.028	0.014	0.028	−0.109

**Table 3:** Sensitivity and specificity for Bayesian group index regression using different imputation methods.

Parameter	Prior Imputation		Sequential Full Bayes		Pseudo-Gibbs		MICE	
10% BDL	Sensitivity	Specificity	Sensitivity	Specificity	Sensitivity	Specificity	Sensitivity	Specificity
exp(β_1_) = 1.00	0.34	0.573	0.33	0.58	0.31	0.575	0.31	0.568
exp(β_2_) = 0.80	0.91	0.797	0.89	0.803	0.9	0.8	0.9	0.8
exp(β_3_) = 1.25	0.82	0.738	0.85	0.753	0.82	0.733	0.84	0.748
exp(β_1_) = 1.00	0.39	0.615	0.38	0.6	0.42	0.623	0.41	0.615
exp(β_2_) = 0.67	0.98	0.943	0.98	0.94	0.98	0.94	0.98	0.94
exp(β_3_) = 1.50	0.99	0.918	1	0.918	1	0.918	0.99	0.92
30% BDL	Sensitivity	Specificity	Sensitivity	Specificity	Sensitivity	Specificity	Sensitivity	Specificity
exp(β_1_) = 1.00	0.28	0.573	0.32	0.56	0.32	0.55	0.29	0.568
exp(β_2_) = 0.80	0.87	0.797	0.89	0.8	0.9	0.8	0.89	0.793
exp(β_3_) = 1.25	0.82	0.705	0.86	0.723	0.84	0.713	0.85	0.703
exp(β_1_) = 1.00	0.38	0.58	0.36	0.593	0.36	0.6	0.4	0.613
exp(β_2_) = 0.67	0.98	0.92	0.97	0.92	0.98	0.927	0.98	0.92
exp(β_3_) = 1.50	0.99	0.893	0.99	0.903	0.99	0.9	0.99	0.903
50% BDL	Sensitivity	Specificity	Sensitivity	Specificity	Sensitivity	Specificity	Sensitivity	Specificity
exp(β_1_) = 1.00	0.38	0.593	0.33	0.593	0.35	0.585	0.37	0.603
exp(β_2_) = 0.80	0.85	0.76	0.81	0.787	0.83	0.8	0.81	0.783
exp(β_3_) = 1.25	0.83	0.705	0.86	0.7	0.83	0.715	0.81	0.703
exp(β_1_) = 1.00	0.38	0.578	0.41	0.605	0.4	0.598	0.41	0.603
exp(β_2_) = 0.67	0.96	0.89	0.98	0.903	0.98	0.903	0.98	0.89
exp(β_3_) = 1.50	0.98	0.87	0.98	0.875	0.99	0.885	0.99	0.873
70% BDL	Sensitivity	Specificity	Sensitivity	Specificity	Sensitivity	Specificity	Sensitivity	Specificity
exp(β_1_) = 1.00	0.32	0.605	0.41	0.62	0.37	0.595	0.37	0.573
exp(β_2_) = 0.80	0.64	0.67	0.72	0.69	0.75	0.693	0.71	0.673
exp(β_3_) = 1.25	0.63	0.675	0.68	0.675	0.74	0.67	0.62	0.66
exp(β_1_) = 1.00	0.39	0.625	0.41	0.62	0.38	0.585	0.4	0.58
exp(β_2_) = 0.67	0.88	0.767	0.87	0.817	0.95	0.79	0.92	0.737
exp(β_3_) = 1.50	0.89	0.775	0.88	0.778	0.89	0.8	0.87	0.743

**Table 4:** Model fit statistics and computation time for Bayesian group index regression using different imputation methods.

Scenario 1	Prior Imputation	Sequential Full Bayes	Pseudo-Gibbs	MICE
10% BDL				
DIC	585.04	585.51	585.53	585.64
pD	5.04	5.03	5.25	5.21
Runtime (min)	7.32	679.71	538.2	7.78
30% BDL				
DIC	585.49	585.58	585.77	585.52
pD	5.39	5.68	5.46	5.1
Runtime (min)	7.31	1567.51	1333.01	7.93
50% BDL				
DIC	585.58	585.56	585.77	586.32
pD	5.15	5.83	6.21	5.52
Runtime (min)	7.03	2375.42	2108.65	8.31
70% BDL				
DIC	587.56	586.25	586.57	588.56
pD	5.05	8.69	9.3	5.59
Runtime (min)	6.33	3557.38	2686.91	9.67
**Scenario 2**	**Prior Imputation**	**Sequential Full Bayes**	**Pseudo-Gibbs**	**MICE**
10% BDL				
DIC	577.71	577.66	577.33	577.57
pD	5.98	6.05	5.7	5.79
Runtime (min)	7.19	683.38	565.97	7.89
30% BDL				
DIC	578.83	578.36	579.46	578.89
pD	6.07	7.08	7.26	5.86
Runtime (min)	7.22	1573.61	1304.99	7.97
50% BDL				
DIC	581.55	580.27	579.18	582.49
pD	6.53	8.01	8.06	6.35
Runtime (min)	6.9	2407.21	2067.7	8.16
70% BDL				
DIC	589.33	586.2	586.11	591.42
pD	5.53	13.42	15.91	6.4
Runtime (min)	6.29	3487.45	2711.79	8.51

**Table 5:** Odds ratio estimates for chemical groups and demographic covariates from the Bayesian group index model (*n* = 583).

Variable	Odds Ratio	2.5% CI	97.5% CI
PCBs	1.19	0.96	1.51
Insecticides	0.64	0.39	1.00
Herbicides	1.17	0.82	1.69
Metals	0.79	0.59	1.06
PAHs	**1.27**	**1.01**	**1.60**
Tobacco	0.82	0.66	1.01
PBDEs	1.21	0.79	1.83
Child’s age	1.01	0.92	1.12
Female	0.98	0.70	1.37
Child’s ethnicity			
Hispanic	1.25	0.81	2.00
Non-Hispanic	1.42	0.91	2.27
Household Income			
$15,000–$29,999	1.02	0.47	2.15
$30,000–$44,999	0.79	0.36	1.61
$45,000–$59,999	0.78	0.34	1.66
$60,000–$74,999	0.45	0.18	1.06
$75,000 or more	**0.38**	**0.17**	**0.79**
Income missing	0.56	0.17	1.61
Mother’s education			
High school	1.25	0.63	2.81
Some college	1.22	0.60	2.84
Bachelor’s or higher	1.21	0.57	2.89
Mother’s age	1.01	0.98	1.05
Residence since birth	**0.66**	**0.44**	**0.96**

Bolded values indicate variables with 95% credible intervals (CI) that do not contain 1.00.

**Table 6:** Odds ratio estimates for chemical groups and demographic covariates from the Bayesian group index model for subjects in highest income bracket (*n* = 266).

Variable	Odds Ratio	2.5% CI	97.5% CI
PCBs	**1.55**	**1.04**	**2.36**
Insecticides	0.51	0.19	1.12
Herbicides	**2.02**	**1.00**	**3.99**
Metals	**0.42**	**0.25**	**0.69**
PAHs	1.19	0.83	1.75
Tobacco	0.77	0.52	1.09
PBDEs	1.12	0.63	2.23
Child’s age	0.98	0.83	1.15
Female	0.70	0.38	1.22
Child’s ethnicity			
Hispanic	1.14	0.47	2.83
Non-Hispanic	1.62	0.87	3.18
Mother’s education			
High school	0.49	0.00	1930.56
Some college	0.20	0.00	730.17
Bachelor’s or higher	0.36	0.00	1375.01
Mother’s age	0.99	0.93	1.05
Residence since birth	**0.40**	**0.21**	**0.76**

Bolded values indicate variables with 95% credible intervals (CI) that do not contain 1.00.

## Data Availability

The CCLS data presented in this study are available on request from the senior author. The data are not publicly available due to privacy restrictions.

## Supplementary Materials

The following are available online at https://www.mdpi.com/article/10.3390/ijerph19031369/s1, Figure S1: Forest plot of chemical group effects for childhood leukemia; Figure S2: Forest plot of chemical group effects for childhood leukemia in children in the highest income bracket; Figure S3: Forest plot of chemical group effects for childhood leukemia in children in the lower income brackets, Table S1: List of chemicals and their group used in the CCLS analyses; Table S2: Odds ratio estimates for chemical groups and demographic covariates from the Bayesian group index model for subjects in lower income brackets.
