# Supplementary material for: Imputation of Below Detection Limit Missing Data in Chemical Mixture Analysis with Bayesian Group Index Regression
Source: Int J Environ Res Public Health. 2022 Jan 26;19(3):1369. doi: 10.3390/ijerph19031369 (PMC8835633; doi:10.3390/ijerph19031369)

Imputation of Below Detection Limit Missing Data with Bayesian Group Index Regression

Matthew Carli[a], Mary H. Ward[b], Catherine Metayer[c], David C. Wheeler[a]

[a]Department of Biostatistics, School of Medicine, Virginia Commonwealth University, Richmond, VA

[b]Occupational and Environmental Epidemiology Branch, Division of Cancer Epidemiology and Genetics, National Cancer Institute, Rockville, MD

[c]UC Berkeley School of Public Health, Berkeley, CA

Table S1: List of chemicals and their group used in the CCLS analyses

Chemical	Chemical Group
PCB-118	PCB
PCB-138	PCB
PCB-153	PCB
PCB-180	PCB
DDE	Insecticide
DDT	Insecticide
Cyfluthrin(I)	Insecticide
Cyfluthrin(II)	Insecticide
Cyfluthrin(III)	Insecticide
Cyfluthrin(IV)	Insecticide
Carbaryl	Insecticide
Propoxur	Insecticide
Pentachlorophenol	Insecticide
gamma-Chlordane	Insecticide
alpha-Chlordane	Insecticide
Chlorpyrifos	Insecticide
Diazinon	Insecticide
Phosmet	Insecticide
cis-Permethrin	Insecticide
Methoxychlor	Insecticide
Cypermethrin(I)	Insecticide
Cypermethrin(II)	Insecticide
Cypermethrin(III)	Insecticide
Cypermethrin(IV)	Insecticide
trans-Permethrin	Insecticide
Piperonyl butoxide	Insecticide
o-Phenylphenol	Herbicide
Trifluralin	Herbicide
Simazine	Herbicide
mCPP	Herbicide
Dicamba	Herbicide
Dacthal	Herbicide
2,4-D	Herbicide
As	Metals
Cr	Metals
Cu	Metals
Pb	Metals
Sn	Metals
W	Metals
Zn	Metals

Indeno(1,2,3-c,d)pyrene	PAH
Dibenz(ah)anthracene	PAH
Dibenzo(ae)pyrene	PAH
Coronene	PAH
Benzo(a)anthracene	PAH
Benzo(a)pyrene	PAH
Benzo(b)fluoranthene	PAH
Nicotine	Tobacco
Cotinine	Tobacco
PBDE-28	PBDE
PBDE-47	PBDE
PBDE-99	PBDE
PBDE-100	PBDE
PBDE-153	PBDE
PBDE-154	PBDE
PBDE-183	PBDE
PBDE-196	PBDE
PBDE-197	PBDE
PBDE-203	PBDE
PBDE-206	PBDE
PBDE-207	PBDE
PBDE-208	PBDE
PBDE-209	PBDE

Figure S1: Forest plot of chemical group effects for childhood leukemia

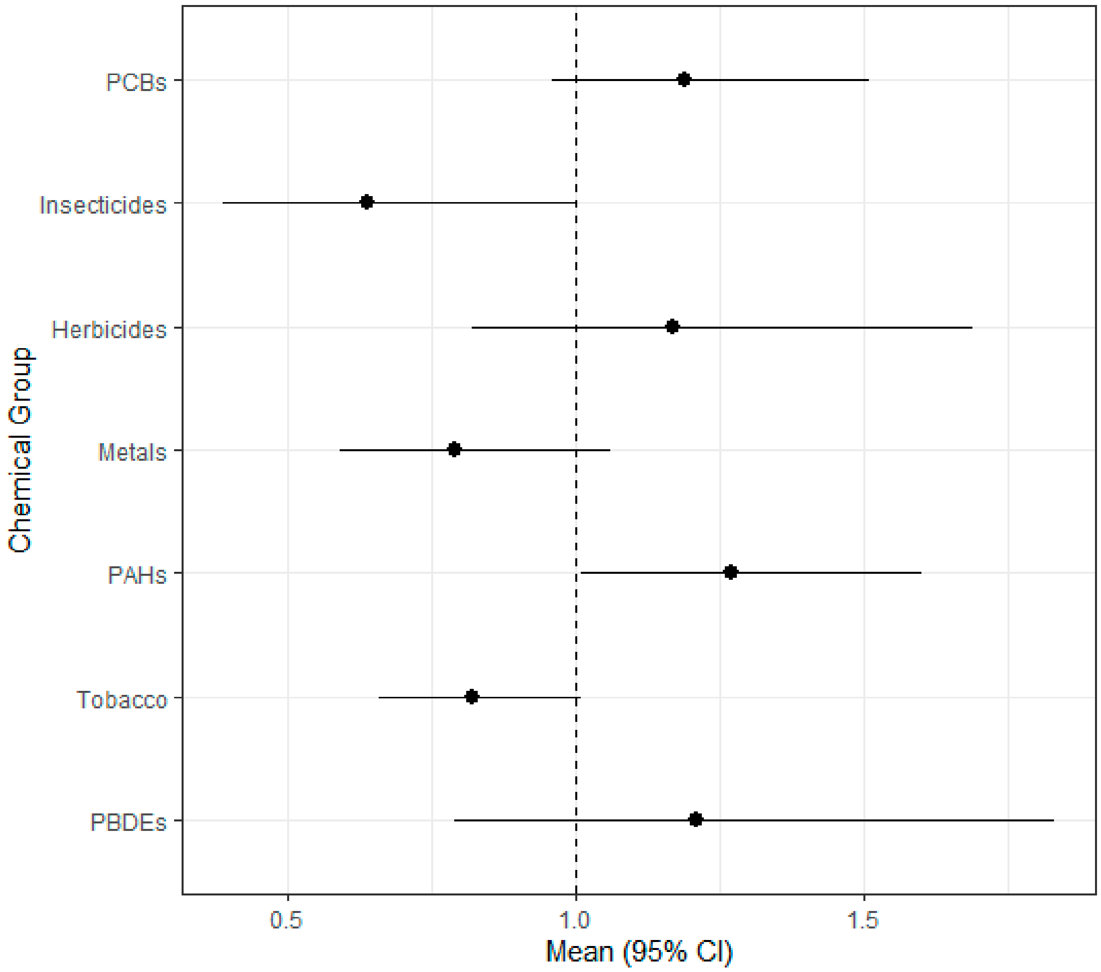

Figure S2: Forest plot of chemical group effects for childhood leukemia in children in the highest income bracket

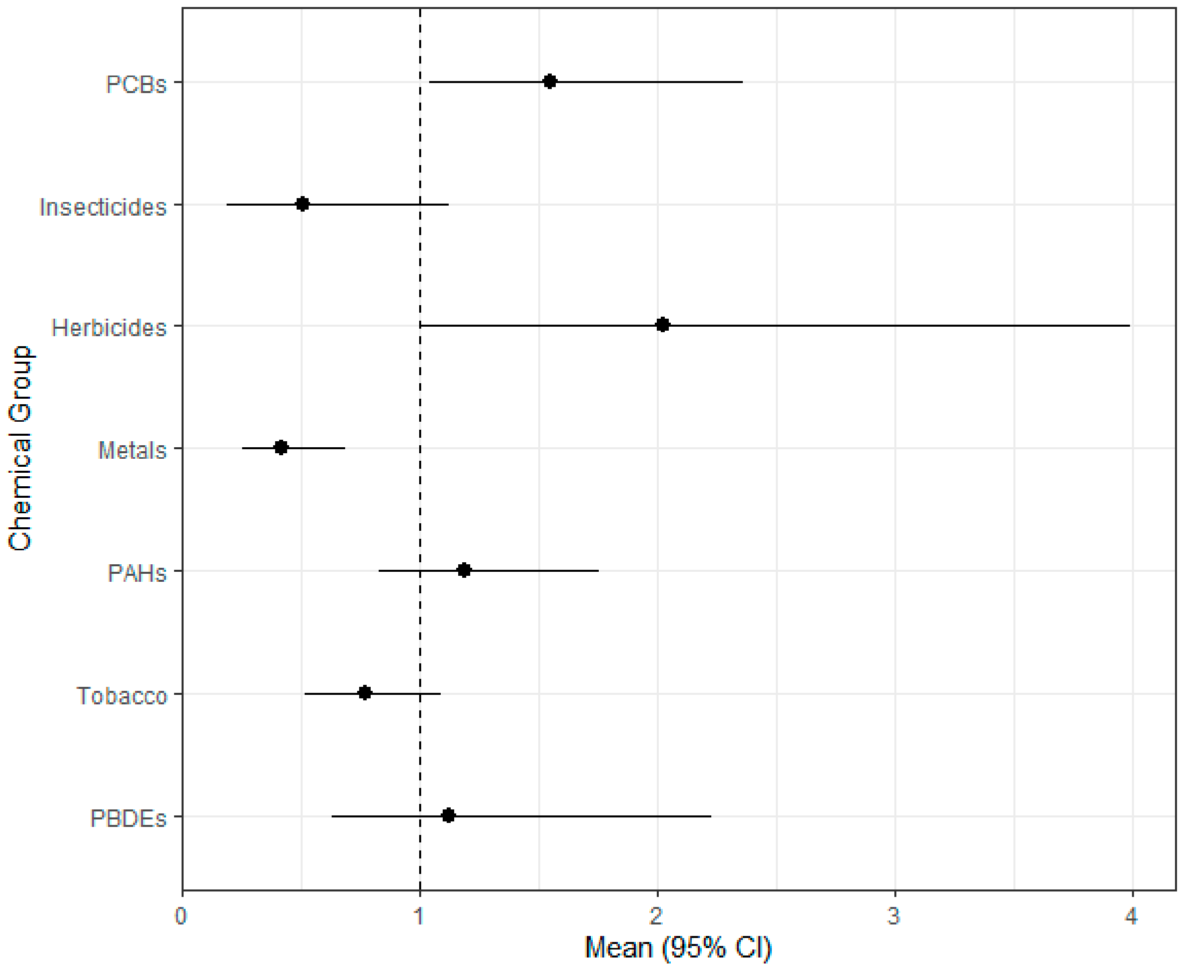

Table S2: Odds ratio estimates for chemical groups and demographic covariates from the Bayesian group index model for subjects in lower income brackets

**Variable**	**Odds Ratio**	**2.5% CI**	**97.5% CI**
PCBs	1.01	0.74	1.38
Insecticides	0.65	0.35	1.10
Herbicides	1.10	0.72	1.78
Metals	1.19	0.80	1.93
PAHs	1.17	0.87	1.62
Tobacco	0.90	0.67	1.19
PBDEs	1.12	0.67	1.89
Child's age	1.03	0.91	1.18
Female	1.40	0.86	2.36
Child's Ethnicity			
Hispanic	1.44	0.83	2.70
Non-Hispanic	1.20	0.59	2.57
Mother's education			
High school	1.32	0.61	3.10
Some college	1.39	0.61	3.35
Bachelor's or higher	0.75	0.29	1.89
Mother's age	1.02	0.98	1.07
Residence since birth	0.94	0.56	1.53

Figure S3: Forest plot of chemical group effects for childhood leukemia in children in the lower income brackets

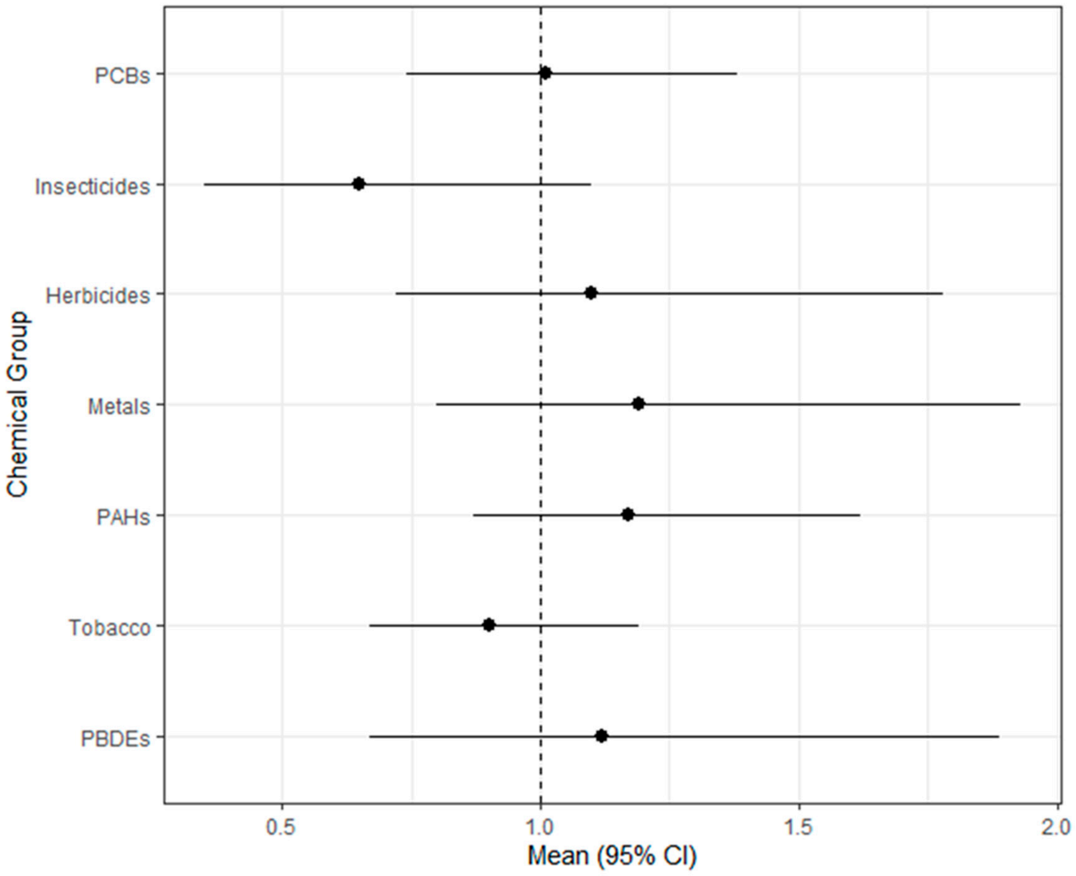

Supplement: Supplementary file 1 [file ijerph-19-01369-s001.zip › ijerph-1525465-supplementary.pdf]
